# Brief exposure to small molecules allows induction of mouse embryonic fibroblasts into neural crest‐like precursors

**DOI:** 10.1002/1873-3468.12572

**Published:** 2017-02-09

**Authors:** Yuzo Takayama, Tamami Wakabayashi, Hiroko Kushige, Yutaka Saito, Yoichiro Shibuya, Shinsuke Shibata, Wado Akamatsu, Hideyuki Okano, Yasuyuki S. Kida

**Affiliations:** ^1^ Biotechnology Research Institute for Drug DiscoveryNational Institute of Advanced Industrial Science and Technology (AIST)TsukubaIbarakiJapan; ^2^ Artificial Intelligence Research CenterNational Institute of Advanced Industrial Science and Technology (AIST)Koto‐kuTokyoJapan; ^3^ Computational Bio Big‐Data Open Innovation Laboratory (CBBD‐OIL)National Institute of Advanced Industrial Science and Technology (AIST)Shinjuku‐kuTokyoJapan; ^4^ Department of Plastic and Reconstructive SurgeryUniversity of TsukubaIbarakiJapan; ^5^ Department of PhysiologyKeio University School of MedicineShinjuku‐kuTokyoJapan; ^6^ Center for Genomic and Regenerative MedicineJuntendo UniversityBunkyo‐kuTokyoJapan

**Keywords:** neural crest, peripheral neuron, small molecules

## Abstract

In this study, we propose a novel method for inducing neuronal cells by briefly exposing them to small‐molecule cocktails in a step‐by‐step manner. Global gene expression analysis with immunohistochemical staining and calcium flux assays reveal the generation of neurons from mouse embryonic fibroblasts. In addition, time‐lapse imaging of neural precursor‐specific enhancer expression and global gene expression analyses show that the neurons are generated by passing through a neural crest‐like precursor stage. Consistent with these results, the neural crest‐like cells are able to differentiate into neural crest lineage cells, such as sympathetic neurons, adipocytes, osteocytes, and smooth muscle cells. Therefore, these results indicate that brief exposure to chemical compounds could expand and induce a substantial multipotent cell population without viral transduction.

## Abbreviations

**CHIR**, CHIR99021

**CNS**, central nervous system

**DM**, dorsomorphin

**EGFP**, enhanced green fluorescent protein

**ES**, embryonic stem

**FSK**, forskolin

**GO**, gene ontology

**iPS**, induced pluripotent stem

**MAP2**, microtubule‐associated protein 2

**MEF**, mouse embryonic fibroblast

**PNS**, peripheral nervous system

**SB**, SB431542

**TH**, tyrosine hydroxylase

**Tranyl**, tranylcypromine

**VPA**, valproic acid

Loss of neuronal cells and nervous system function resulting from injury or neurodegenerative diseases, such as Alzheimer's disease 1, amyotrophic lateral sclerosis 2, and familial dysautonomia 3, diabetes 4, Guillain–Barrë syndrome 5, Hirschsprung disease 6, may cause severe cognitive and motor disabilities owing to the limited proliferative and regenerative potential of neuronal cells. Therefore, the development of regenerative methods is important for the recovery of nervous system function and for investigation and clarification of the progressive mechanisms of diseases.

Neural crest cells differentiate into neuronal cells found in the region outside the brain and spinal cord as peripheral neurons, including sensory, enteric, sympathetic, and parasympathetic neurons. These neural crest cells also give rise to glial Schwann cells, melanocytes, endocrine cells, smooth muscle cells, and peripheral neurons. Additionally, in bone marrow or adipose tissues, neural crest cells are the source of mesenchymal stem cells that can differentiate into cartilage, bone, and white adipocytes. The multipotent phenotype and self‐renewal capacity of these cells are expected to have potential applications in cell‐based therapies 7. In addition, neural crest cells are related to serious diseases, such as Hansen disease, in which *Mycobacterium leprae* initially infects Schwann cells, which later dedifferentiate into progenitor/stem‐like cells, causing symptoms of granuloma formation, skin lesions, and peripheral nervous system (PNS) damage, including sensory loss.

Treatment with small molecules, such as regulators of specific signaling pathways or epigenetic states, has recently attracted much attention for applications in cell differentiation and induction. Notably, Hou *et al*. reported that mouse pluripotent stem cells could be generated from somatic cells using only small molecules 8. These studies have suggested that optimization of small‐molecule combinations may facilitate the generation of neuronal cells from neuronal stem cells and non‐neuronal cells.

In this study, we proposed a method for production of peripheral neurons and other neural crest‐derived cells using only small‐molecule compounds. Our results suggested that brief exposure to chemical compounds in a step‐by‐step manner facilitated rapid cell induction for acquisition of murine multipotent neural crest‐like precursors.

## Materials and methods

### Cell culture

All the procedures were performed in accordance with the guidelines of the National Institute of Advanced Industrial Science and Technology (AIST) Animal Care and Use Committee (approval no.: 2013‐186).

Mouse embryonic fibroblasts (MEFs) were isolated from 12.5‐day‐old embryos of C57BL/6NCrSlc mice. Cells were cultured in Dulbecco's modified Eagle's medium (DMEM) supplemented with 20% fetal bovine serum (FBS), 1% nonessential amino acids (NEAAs), and 1% penicillin/streptomycin (PS); these cells were defined as P0 MEFs. To assess the effects of small molecules, we used embryonic fibroblasts from two strains of transgenic mice in which neural and neural crest‐derived cells specifically expressed enhanced green fluorescent protein (EGFP) under the control of the promoter/enhancer of the *Nestin* gene (Nestin‐EGFP MEFs 9) and under the control of the CAG‐CAT‐EGFP reporter specifically induced by expression of the *Wnt1* promoter‐driven Cre recombinase gene (Wnt1‐Cre/EGFP MEFs 10, 11, 12), respectively. These transgenic strains were supplied by Shinsuke Shibata, Wado Akamatsu, and Hideyuki Okano.

### Chemical treatment

To modulate cellular fate, small molecules were applied to the cultured samples. A combination of 0.25 mm valproic acid (VPA; Wako Pure Chemical Industries, Osaka, Japan), 1.5 μm CHIR99021 (CHIR; Cayman Chemical Company, Ann Arbor, MI, USA), 0.5 μm 616452 (Merck Millipore, Darmstadt, Germany), 5 μm forskolin (FSK; Wako Pure Chemical Industries), and 2.5 μm tranylcypromine (Tranyl; Abcam, Cambridge, UK) was used. The cocktail was mixed in DMEM supplemented with 20% FBS, 1% NEAAs, 1% PS, 50 ng·mL^−1^ basic fibroblast growth factor (bFGF), and culture supernatants from CHO cells producing leukemia inhibitory factor (LIF), a kind gift from T. Nakano (Osaka University, Japan). The chemical medium was applied to the culture samples for 2 days. Another small‐molecule cocktail, composed of 0.25 mm VPA, 5 μm FSK, 2.5 μm Tranyl, 2.5 μm dorsomorphin (DM; Sigma‐Aldrich, St. Louis, MO, USA), and 2.5 μm SB431542 (SB; Sigma‐Aldrich), was then applied for 1 day. This small‐molecule cocktail was also mixed in DMEM‐based medium as described above.

### Neuronal differentiation

After exposure to the small‐molecule cocktails, cells were cultured in differentiation medium consisting of DMEM/F‐12 (Wako Pure Chemical Industries), 25 μg·mL^−1^ insulin (Wako Pure Chemical Industries), 50 μg·mL^−1^ human transferrin (Sigma‐Aldrich), 30 nm sodium selenite (Sigma‐Aldrich), 20 nm progesterone (Sigma‐Aldrich), 100 nm putrescine (Sigma‐Aldrich), and 1% PS. For neuronal differentiation, the differentiation medium was further supplemented with 1 μm retinoic acid (RA; Sigma‐Aldrich), 10 μm FSK, 10 ng·mL^−1^ brain‐derived neurotrophic factor (BDNF; Wako Pure Chemical Industries), 10 ng·mL^−1^ glial cell‐derived neurotrophic factor (GDNF; Wako Pure Chemical Industries), and 50 μg·mL^−1^ ascorbic acid (Wako Pure Chemical Industries); this was defined as the neuronal differentiation medium (NDM). Before adding cells, the culture dishes were coated with 20 μg·mL^−1^ poly‐l‐ornithine (PLO; Sigma‐Aldrich) for 1 h at 37 °C, followed by treatment with 5 μg·mL^−1^ laminin (Sigma‐Aldrich) for 2 h at 37 °C. The cells were then seeded onto the coated dishes at a density of 5 × 10^4^ cells·cm^−2^. Half of the NDM was exchanged twice a week to maintain the cultures.

### Time‐lapse imaging

Time‐lapse fluorescent microscopy was performed using an IncuCyte ZOOM imaging system (Essen BioScience, Ann Arbor, MI, USA). Nestin‐EGFP MEFs were plated onto 0.1% gelatin‐coated 24‐well plates (Corning, NY, USA) at a density of 5 × 10^4^ cells·well^−1^. Medium was changed to the chemical medium the next day and then again after 3 days, as described above, using chem(all) conditions. For assessment and comparisons, samples with other chemical combinations [V, T, or chem(‐F)] and a control condition (without chemicals) were prepared. Fluorescent images were sequentially captured every 30 min for 70 h after applying the chemical medium. The captured area was 1.7 × 1.27 mm.

Wnt1‐Cre/EGFP MEFs were plated onto 0.1% gelatin‐coated 96‐well plates (Corning) at a density of 1 × 10^4^ cells·well^−1^. The next day, Wnt1‐Cre/EGFP MEFs were treated with small molecules in a step‐by‐step chemical treatment as described above. As a control, Wnt1‐Cre/EGFP MEFs were cultured with DMEM supplemented with 10% FBS, 1% NEAAs, and 1% PS for 3 days. To confirm the induction of neural crest‐like precursors from MEFs, these cells were replaced with neural crest maintenance medium for 1 week; this medium was comprised of the differentiation medium supplemented with 10 ng·mL^−1^ bFGF and 20 ng·mL^−1^ epidermal growth factor (EGF; Wako Pure Chemical Industries) 13. EGFP expression was monitored by fluorescence microscopy every day during the chemical treatment for the first 3 days and at day 10.

### Adipocyte differentiation

To induce adipocyte differentiation in chemical‐treated cells, the chemical medium on day 4 was replaced with differentiation medium supplemented with 10 μm FSK and 5 μm SB. The differentiation medium was changed every 2 days.

### Osteocyte differentiation

To induce osteocyte differentiation in the chemical‐treated cells, the chemical medium on day 4 was replaced with StemPro Osteogenesis differentiation medium (Thermo Fisher Scientific, Waltham, MA, USA). The differentiation medium was changed every 2 days. To confirm osteocyte differentiation, the samples were stained with Alizarin red solution (Wako Pure Chemical Industries). Briefly, the samples were fixed with 3.7% formaldehyde (Wako Pure Chemical Industries). After washing with distilled water four times, the samples were stained with 2% Alizarin red solution. The samples were then washed with distilled water four times and observed.

### Smooth muscle differentiation

To induce smooth muscle differentiation in the chemical‐treated cells, the chemical medium on day 4 was replaced with DMEM supplemented with 10% FBS, 1% NEAAs, 1% PS, and 10 ng·mL^−1^ transforming growth factor (TGF)‐β1 (Peprotech, Rocky Hill, NJ, USA). The differentiation medium was changed twice a week.

### Quantitative real‐time reverse transcription polymerase chain reaction (RT‐qPCR)

Total RNA was isolated from cells using a ReliaPrep RNA Cell Miniprep system (Promega, Madison, WI, USA). The purity and concentration of RNA were determined using a NanoDrop Lite spectrophotometer (Thermo Fisher Scientific). One‐hundred nanograms of total RNA was reverse transcribed to cDNA with a ReverTra Ace qPCR RT Kit (Toyobo, Osaka, Japan). RT‐qPCR was then performed using a 7900HT Fast Real‐Time polymerase chain reaction (PCR) System (Applied Biosystems, Foster City, CA, USA) with Thunderbird SYBR qPCR Mix (Toyobo). The expression values were normalized to *U36b4* expression and are shown as means ± standard deviations (SDs) of triplicate measurements. The primer sequences are listed in Table S1.

### Microarray analysis

**Figure 1:**
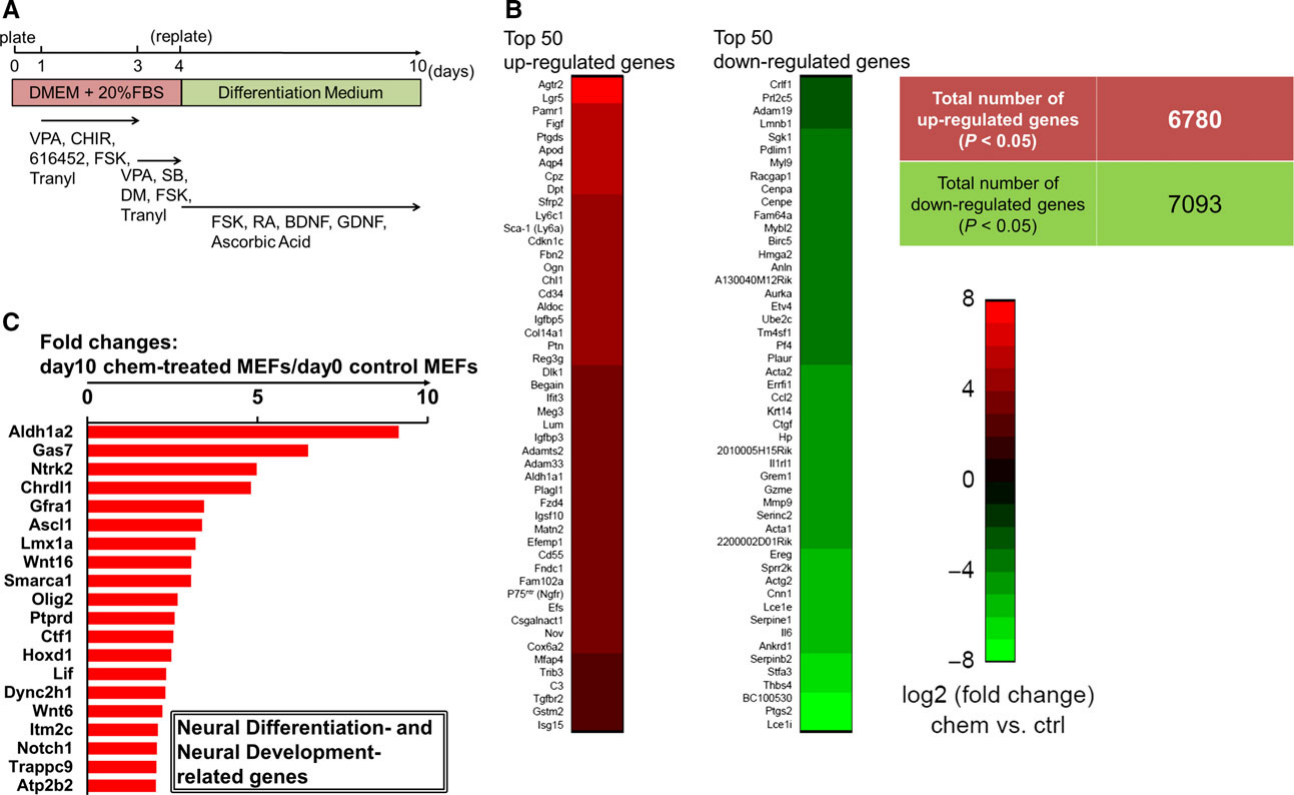
Microarray analysis of MEFs treated with small‐molecule compounds. (A) Scheme of the step‐by‐step induction method with small‐molecule cocktails. (B) Graphs of the top 50 upregulated and downregulated genes in the chemical‐treated sample (day 10) relative to those in the control sample. Upregulated and downregulated genes with *P* values of < 0.05 were sorted according to fold changes in gene expression levels. The total numbers of upregulated and downregulated genes are also shown. (C) Graph of the top 20 upregulated neural differentiation‐ and neural development‐related genes in chemical‐treated cells (day 10) relative to those in the control sample. Upregulated genes with *P* values of < 0.05 were sorted by fold change in gene expression.

Total RNA extracted from chemical‐treated MEFs and control MEFs on day 10 was used for microarray analysis. Total RNA (140 ng) was reverse transcribed and labeled with Cy3 using a Low Input Quick Amp Labelling Kit, One Color (Agilent Technologies, Tokyo, Japan) according to the manufacturer's protocol. Hybridization to the microarrays was performed using a Gene Expression Hybridization Kit (Agilent Technologies) according to the manufacturer's protocol. Expression profiling was performed with a SurePrint G3 Mouse GE microarray 8 × 60 K (Agilent Technologies). An Agilent DNA microarray scanner and Feature Extraction Software were used to determine the fluorescence intensities of spots on the microarray. Samples were run in triplicate. The obtained data were intra‐ and interarray normalized, and differentially expressed genes with *P* values of < 0.05 were detected using the limma package 14 in the R statistical computing environment. Gene ontology (GO) and KEGG pathway enrichment analyses were performed in the Database for Annotation, Visualization, and Integrated Discovery (DAVID) 15. Biological process GO terms having *P* values of < 0.01 and KEGG pathways having *P* values of < 0.05 were considered significant. All *P* values in GO and KEGG pathway enrichment analyses and differential expression analysis were corrected for multiple testing with the Benjamini–Hochberg method. The list of neural crest‐related genes used in Fig. 3G was extracted from the GO database (16; GO:0014032 and GO:0014033) with several genes added from the literature 17, 18. The list of neuron‐related genes used in Fig. 1C was also obtained from the GO database (GO:0030182 and GO:0048666). The microarray data were deposited in the NCBI Gene Expression Omnibus database, with accession number GSE78890 (http://www.ncbi.nlm.nih.gov/geo/query/acc.cgi?token=atijcawifrubdqd&acc=GSE78890).

### Immunohistochemical analysis

Immunohistochemical staining was performed as previously described 19. Primary antibodies were added and incubated overnight at 4 °C. The next day, cells were washed three times with 0.2% Tween‐20 and then incubated for 2 h at room temperature with secondary antibodies (anti‐mouse Alexa Fluor‐488 and anti‐rabbit Alexa Fluor‐555 [1 : 1000; Thermo Fisher Scientific]). The primary antibodies used in this study were as follows: mouse anti‐β‐III‐tubulin (TUJ1; 1 : 3000; Covance, Richmond, CA, USA 20), mouse anti‐microtubule‐associated protein 2 (MAP2; 1 : 1000; Abcam 20), rabbit anti‐NeuN (1 : 1000; Merck Millipore 20), rabbit anti‐tyrosine hydroxylase (TH; 1 : 500; Merck Millipore 21), rabbit anti‐synapsin‐1 (1 : 1000; Merck Millipore 20), rabbit anti‐peripherin (1 : 1000; Merck Millipore 22), rabbit anti‐α‐smooth muscle actin (SMA; 1 : 100; Abcam 23), and rabbit anti‐SRY‐box 10 (SOX10; 1 : 200; Abcam 24). For nuclear staining, 0.2 μg·mL^−1^ Hoechst 33342 (Dojindo Molecular Technologies, Kumamoto, Japan) was also added.

### Calcium imaging

Chemical‐treated MEFs were functionally examined using calcium imaging. Samples were labeled with 5 μg·mL^−1^ fluo‐4/AM (Thermo Fisher Scientific) for 30 min. After dye labeling, the medium was replaced with Ringer's solution (148 mm NaCl, 2.8 mm KCl, 2 mm CaCl_2_, 1 mm MgCl_2_, 10 mm HEPES, and 10 mm glucose; pH 7.4). Fluorescence was detected using an EM CCD camera and Metamorph software (*n* = 10 cells for each condition). For stimulation of neuronal cells, a high potassium solution (101.8 mm NaCl, 50 mm KCl, 2 mm CaCl_2_, 1 mm MgCl_2_, 10 mm HEPES, and 10 mm glucose; pH 7.4) was used. A frame rate of 2 frames·s^−1^ was used. The recorded signals were analyzed with imagej (National Institutes of Health; available at http://imagej.nih.gov/ij/). For detection of calcium spikes, a threshold of 0.1 for Δ*F*/*F* was used.

### Statistical analysis

All data are expressed as means ± SDs. For qPCR and calcium imaging experiments, statistical analyses were performed using two‐tailed unpaired *t*‐tests. For microarray experiments, statistical analyses were carried out as described above. Differences with *P* values of < 0.05 were considered statistically significant.

## Results

For application of small‐molecule compounds, we selected seven small molecules based on currently available reprogramming methods 8: VPA, CHIR, 616452, FSK, Tranyl, DM, and SB. VPA is a histone deacetylase inhibitor that improves cell reprogramming efficiency by combining with transcription factors 25, whereas CHIR is a glycogen synthase kinase 3 (GSK3) inhibitor that activates Wnt signaling and improves cell reprogramming efficiency 26. 616452, an inhibitor of TGF‐β type 1 activin‐like kinase receptor (ALK) 5, can replace the transcription factor SOX2 and enhance NANOG expression 27. FSK is a cAMP signaling activator that potentiates neuronal differentiation 28. Additionally, both FSK and Tranyl, a lysine‐specific demethylase 1 inhibitor, improve cell reprogramming efficiency 29. Finally, DM is a bone morphogenic protein (BMP) signaling inhibitor, and SB is an inhibitor of TGF‐β type 1 ALK4, ALK5, and ALK7. The combination of DM and SB can efficiently induce neuronal differentiation in human embryonic stem (ES) and induced pluripotent stem (iPS) cells 30. In this study, we treated the cells with a combination of VPA, CHIR, 616452, FSK, and Tranyl (VC6FT) for 2 days and a combination of VPA, DM, SB, FSK, and Tranyl (VDSFT) for an additional 1 day, followed by differentiation into neuronal cells in differentiation medium (Fig. 1A).

We first performed a comparative gene expression analysis of chemical‐treated and control MEFs using global gene expression profiles measured by microarrays. From a global point of view, a total of 6780 upregulated and 7093 downregulated genes were identified (*P* < 0.05; Fig. 1B). Interestingly, the top 50 upregulated genes included neuron‐associated genes, such as *Agtr2*,* Lgr5*,* Ptgds*, and *Aqp4*. In contrast, the top 50 downregulated genes included cell cycle‐ and proliferation‐associated genes, such as *Ptgs2* and *Thbs4*, and the fibroblast‐associated *Acta2* gene. Furthermore, microarray‐based transcriptome analysis confirmed the upregulation of various neural differentiation‐ and development‐related markers (Fig. 1C). Thus, the small molecule‐based culture method strongly affected cell identity and specifically induced neural differentiation and development‐related genes.

### Neural differentiation from chemical‐treated MEFs

**Figure 2:**
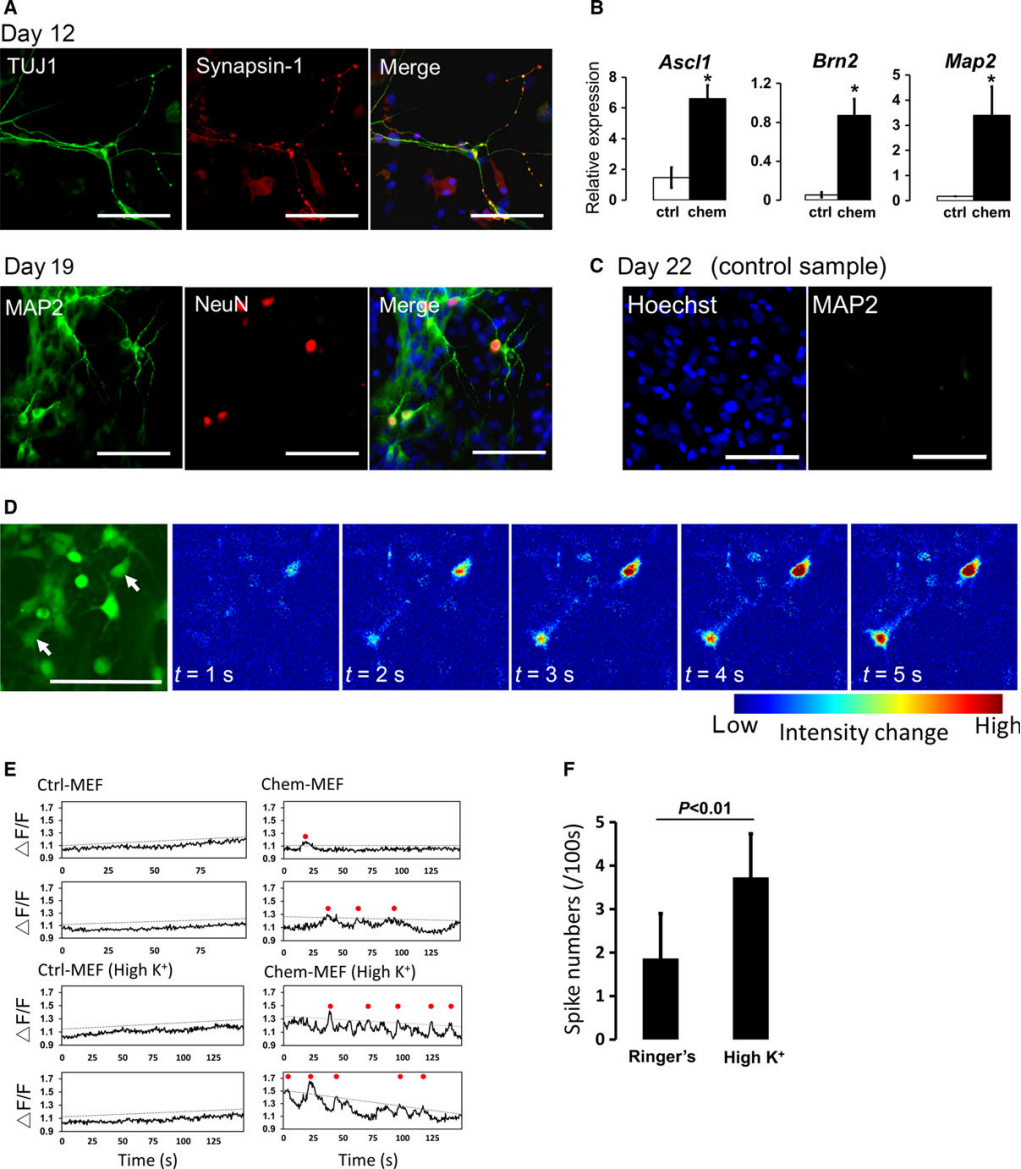
Generation of neuronal cells by brief exposure to small‐molecule compounds. Immunohistochemical staining of chemical‐treated MEFs showing TUJ1 and synapsin‐1 on day 12 and MAP2 and NeuN on day 19 (A). (B) qPCR analysis of *Ascl1*,* Brn2*, and *Map2* genes in control and chemical‐treated cells on days 12 and 13. The expression value of each target gene was normalized to that of *U36b4*. (C) MAP2‐positive cells were not detected in control samples. The day 4 MEFs (without chemicals) were transferred to PLO/laminin‐coated plates and cultured in neural differentiation medium until day 22. (D) Spontaneous calcium transients in the induced neuronal cells on day 16. Left: the induced neuronal cells labeled with the calcium indicator fluo‐4. Right: fluorescent intensities in the left panel. Two independent neuronal cells (marked with white arrows) exhibited spontaneous calcium transients. (E) Typical traces of intracellular calcium transients in the control and induced neuronal cells. Traces of two cells for each condition are presented. The induced neuronal cells showed distinct calcium activities that could be enhanced by application of concentrated potassium saline. Dotted lines and red circles indicate calculated thresholds for each trace and calcium spikes exceeding each threshold, respectively. (F) Bar graphs show the effects of applying 50 mm potassium saline to the induced neuronal cells. Scale bar: 100 μm. *P < 0.05.

Next, we attempted to identify the neuronal differentiation capability of the chemical‐treated MEFs. As expected, these cells were positive for TUJ1, synapsin‐1, MAP2, and NeuN at several days after treatment (Fig. 2A), indicating that the chemical treatment protocol caused induction of neuronal cells. In addition, RT‐qPCR analysis showed that the expression levels of typical marker genes for neuronal lineages, such as *Ascl1*,* Brn2*, and *Map2*, were significantly elevated in the induced cells, in contrast with those in untreated cells (Fig. 2B). Incidentally, there were no MAP2‐positive neurons generated without chemical treatment from control MEFs (Fig. 2C).

We also confirmed the neuronal activities of the generated cells using calcium imaging. Notably, no distinct spontaneous activity was detected in the control samples (Movie S1). In contrast, spontaneous calcium transients with onset and decay occurring within 20 s were observed in the chemical‐treated cells (Fig. 2D, Movie S2). Traces of calcium transients in selected cells (arrows in Fig. 2D) are shown in Fig. 2E. Analysis of the calcium activity in the chemical‐treated cells revealed significantly increased activity following application of 50 mm potassium (High K^+^) saline solution compared with that in Ringer's solution (Fig. 2E,F). Notably, the induced neurons exhibited spontaneous calcium activity having a certain duration, which was relatively slow for central nervous system (CNS) neurons (within several seconds), but consistent with PNS neurons 31. Thus, the generated neurons were thought to be peripheral neurons.

### Chemical compounds induced the transdifferentiation of MEFs into neural crest‐like lineages

**Figure 3:**
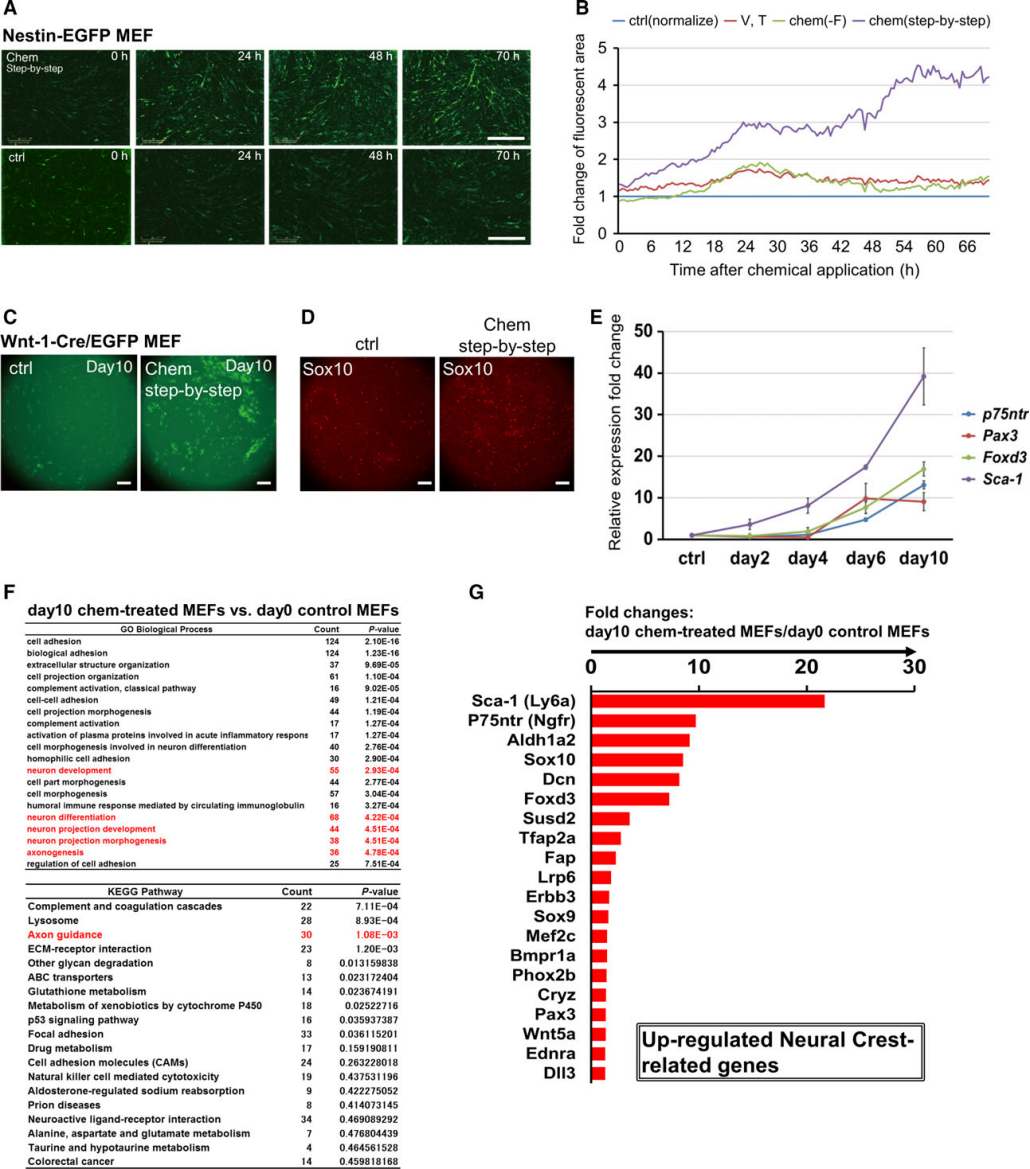
Analysis of small‐molecule compound‐based neural crest‐like precursor induction processes. (A) Time‐lapse fluorescent image series of GFP fluorescence derived from the Nestin‐EGFP MEFs in control (without small‐molecule treatment) and chemical‐treated samples. (B) Time series analysis of Nestin‐EGFP MEFs with application of several small‐molecule treatment patterns. Fold changes in the fluorescent area in chemical‐treated cells during a 70‐h period are shown. The Nestin‐EGFP MEFs were exposed to four chemical combinations [ctrl: without chemicals; V, T: VPA and Tranyl; chem(‐F): VPA, CHIR, 616452, and Tranyl; chem (step‐by‐step)]. Time courses of the GFP‐positive areas in the three chemical conditions were normalized to that of the control condition. (C) Fluorescent images of GFP fluorescence derived from the Wnt1‐Cre/EGFP MEFs in control (without small‐molecule treatment) and chemical‐treated samples at day 10. (D) Increase in SOX10‐positive cells (red) in the chemical‐treated samples in contrast to the control samples on day 10. The control samples were maintained in DMEM‐based medium for the first 4 days and then in neural crest maintenance medium until day 10. (E) Time series changes in the expression levels of neural crest‐related genes in chemical‐treated MEFs. The expression level of each target gene was normalized to that of the control sample. (F) Functional annotation of upregulated genes in the chemical‐treated sample (day 10) relative to those in the control sample by GO and KEGG pathway analysis. Top 20 gene categories, gene numbers, and *P* values are shown. Red font indicates neural differentiation‐ and development‐related categories. (G) Graph of the top 20 upregulated neural crest‐related genes in the chemical‐treated cells (day 10) relative to those in the control sample. Upregulated genes with *P* values of < 0.05 were sorted by fold change in gene expression. Scale bar: 100 μm.

To investigate the induction process involved in neuron generation, we focused on marker gene expression during this process. The *Nestin* gene is known to be expressed in neural progenitor cells and neural crest stem cells 32, 33. Therefore, we first examined changes in EGFP fluorescence in MEFs that specifically expressed EGFP under the control of the neural promoter/enhancer of the *Nestin* gene (Nestin‐EGFP MEFs, 9; Fig. 3A). The area of EGFP‐positive cells and the intensity of the fluorescence were significantly increased after 70 h in chemical‐treated cells. Notably, MEFs intrinsically include a small population of EGFP‐positive cells (0.5–1%). After chemical treatment, EGFP‐positive cells increased to 3.7% ± 0.7%. Analysis of video recordings showed that the increased fluorescence could be explained by proliferation of EGFP‐positive cells, elevation of EGFP expression levels, and the new appearance of EGFP‐positive cells (Movies S3 and S4). We also analyzed changes in fluorescence with several treatment patterns, as summarized in Fig. 3B. The fluorescent area of chemical‐treated samples was normalized to that of the control sample at each time point, allowing us to determine the fold change at each time point. Notably, the EGFP‐positive regions gradually increased to more than four times the area of the control. However, when the MEFs were exposed to other chemical combinations, such as V, T (VPA and Tranyl), or chem(‐F) (VPA, CHIR, 616452, and Tranyl) for 70 h, distinct increases in EGFP‐positive cells were not observed. These data indicated that our proposed chemical pattern effectively induced *Nestin*‐positive neural stem cells or neural crest cells during the early stages of cell fate determination.

Moreover, we monitored EGFP fluorescence using MEFs derived from double transgenic mice harboring EGFP with *Wnt1* gene regulatory regions (Wnt1‐Cre/EGFP MEFs) 7, 8, 9 to examine whether the induced cells were neural crest cells. The Wnt signaling pathway is involved in early specification of SOX10‐ or paired box 3 (PAX3)‐positive neural crest cells during the embryonic stage 24. Similar to those in Nestin‐EGFP MEFs, Wnt1‐Cre/EGFP MEFs also intrinsically include a small population of EGFP‐positive cells. Although the number of EGFP‐positive cells and the intensity of pre‐existing EGFP‐positive cells were the same as those in controls during the 72‐h chemical treatment, the fluorescence was dramatically increased after continuous culture in neural crest maintenance medium at day 10 (Fig. 3C). After the chemical treatment, the rate of EGFP‐positive cells increased to 9.4% ± 4.5%. To evaluate whether the induced cells were neural crest‐like precursors, we examined SOX10 expression by immunohistochemical staining. The number of SOX10‐positive cells increased about twofold in step‐by‐step chemical treatment compared with that in the control samples (Fig. 3D). We also examined double staining of Wnt1 and SOX10 and confirmed that about 40% of EGFP‐positive cells were positive for SOX10 (Fig. S3). These results indicated that the chemical treatment drove *Nestin* gene expression, induced *Wnt1* gene expression, and caused MEFs to develop into SOX10‐positive neural crest‐like precursors.

As described above, Nestin‐EGFP and Wnt1‐Cre/EGFP MEFs intrinsically included a small population of weakly EGFP‐positive cells at the start of each experiment. Moreover, the induced neural crest‐like cells already lost the capacity for self‐renewability in the conventional neural crest maintenance medium (data not shown), showing downregulation of cell cycle‐ and proliferation‐associated genes, such as *Ptgs2* and *Thbs4* (Fig. 1B). Thus, these data suggested that the positive expression status of some of neural crest markers, such as Sca‐1, P75NTR, and Sox10, in response to our chemical treatment indicated the transient move toward the neuronal differentiation.

Based on the above results, we performed a time series RT‐qPCR profiling experiment using the induced cells on days 0 (as a control), 2, 4, 6, and 10 with step‐by‐step chemical treatment. The expression levels of neural crest markers, including *P75* ^*ntr*^ (also known as *Ngfr*), *Pax3*,* Foxd3*, and *Sca‐1* (also known as *Ly6a*) 23, 34, were significantly elevated (Fig. 3E and Fig. S1). Interestingly, the expression levels of CNS neural stem cell markers, including *Pax6* and *Sox2* 35, were not elevated during the chemical treatment (Fig. S2). Additionally, GO and KEGG pathway enrichment analyses for upregulated genes measured by microarrays are shown in Fig. 3F. These analyses revealed that several affected terms and pathways were related to neural differentiation and development. Specifically, microarray‐based transcriptome analysis confirmed the upregulation of various neural crest‐related markers (Fig. 3G). Taken together, our data demonstrated that the step‐by‐step protocol induced a fraction of neural crest‐like precursors in MEFs.

### Chemical‐treated MEFs differentiated into neural crest derivatives

**Figure 4:**
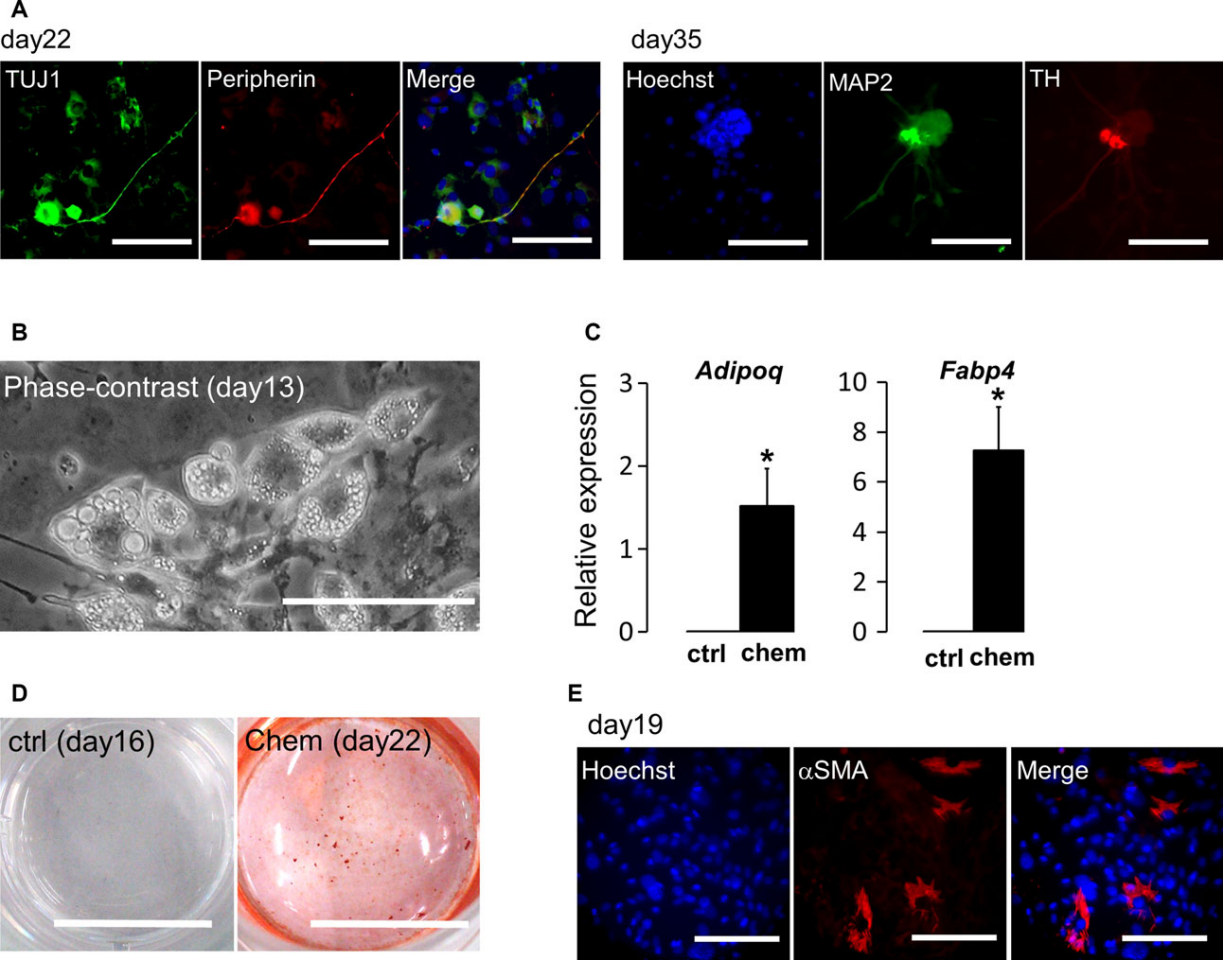
Differentiation from neural crest‐like precursors. (A) Induction of peripheral neurons was confirmed by immunohistochemical staining for TUJ1, peripherin, and TH. Scale bar: 100 μm. (B) Adipocyte differentiation was confirmed by cell morphology (including the presence of lipid‐like droplets) and marker gene expression. (C) Analysis of the expression levels of adipocyte marker genes (*Adipoq* and *Fabp4*). Scale bar: 100 μm. (D) Osteocyte differentiation was confirmed by Alizarin red staining. Alizarin red‐positive cells were detected in chemical‐treated cells on day 22. Scale bar: 10 mm. (E) Smooth muscle differentiation was confirmed by immunohistochemical staining for αSMA in chemical‐treated cells on day 19. Scale bar: 100 μm. *P < 0.05.

To confirm the differentiation capacity of the induced neural crest‐like precursors, we examined whether the induced cells could differentiate into peripheral neurons, adipocytes, osteocytes, and smooth muscle cells, which are characteristic lineages of neural crest‐derived cells 23. As expected, most TUJ1‐ or MAP2‐positive cells coexpressed peripherin (Fig. 4A), indicating that induced neurons were predominantly peripheral neurons. Interestingly, several MAP2‐positive cells coexpressed the gene encoding TH, which is a marker of sympathetic neurons in peripheral neurons on day 35 (right panels in Fig. 4A). Additionally, after 1 week of culture in adipose differentiation medium, lipid droplet‐like structures were observed (Fig. 4B), and the expression levels of the adipocyte marker (*Adipoq*) and fatty‐acid‐binding protein 4 (*Fabp4*) were elevated (Fig. 4C). For osteocyte differentiation, differentiated osteocytes were monitored by Alizarin red staining (Fig. 4D). Finally, smooth muscle cell differentiation was observed by analysis of αSMA expression (Fig. 4E). Taken together, these findings suggested that step‐by‐step chemical treatment rapidly and transiently induced neural crest‐like precursors.

## Discussion

In this study, we evaluated the mechanisms through which neural crest‐like cells are induced following exposure to the proposed step‐by‐step chemical cocktails. Hou *et al*. reported that the combination of VPA, CHIR, 616452, FSK, and Tranyl significantly induced pluripotency‐related genes in fibroblasts 8, suggesting that brief exposure to these small‐molecule cocktails induced partial reprogramming of cells. However, the combination of a GSK3 inhibitor, a TGF‐β/activin/nodal inhibitor, and a BMP inhibitor has been shown to be useful for induction of neural crest lineage cells from ES or iPS cells 17, 36. In these reports, CHIR and (2′Z,3′E)‐6‐bromoindirubin‐3′‐oxime (BIO) were used as GSK3 inhibitors, SB was used as an inhibitor of TGF‐β/activin/nodal, and LDN‐193189 and Noggin were used as BMP inhibitors. For the small molecules used in this paper, VPA, FSK, and Tranyl are thought to function in partial reprogramming or as a supportive reagent 25, 28, 29. Furthermore, other small molecules (the GSK3 inhibitor CHIR, the BMP inhibitor DM, and the TGF‐β/activin/nodal inhibitor SB) are used for inducing NCCs from ES or iPS cells, as described above. Based on this knowledge, we believe that our chemical treatment protocol partially reverted MEFs to a pluripotent state by affecting VPA, FSK, and Tranyl; additionally, the regulation of cellular fate to the neural crest lineage was affected by CHIR, DM, and SB. Moreover, our proposed culture conditions may simultaneously facilitate selective expansion and proliferation of naïve neural crest cells that are intrinsically present in the obtained tissues. In either case, our proposed protocol is useful for obtaining neural crest‐like precursors and their derivatives.

Gene delivery techniques are commonly used for cell reprogramming and conversion. However, the drawbacks of viral transduction, including integration of exogenous genes into the target cells, may limit the application of virus‐based methods in therapeutic approaches. Here, we proposed a method for treatment of cells with small‐molecule cocktails and confirmed the generation of peripheral neuronal cells from somatic cells. Although small molecule‐based induction of specific cell types has been recently reported for neurons 37, 38, 39, pancreatic cells 40, and Schwann cells 41, induction of neural crest‐like precursors and its derivatives have only been reported using a *Sox10* gene‐transfer method 42. One of the most important features of step‐by‐step chemical treatment is the rapid treatment protocol. We could induce MAP2‐positive neuronal cells from somatic cells within 10 days, in contrast to chemically iPS cells, which required over 1 month 8. Rapid production of neurons is a key factor required for cell transplantation and regenerative medicine and is necessary to avoid inflammation, which may induce glial scars and inhibit neuroregeneration 43, 44. On the other hand, the induction rate in the experiments is rather low efficiency for further application. Differentiation, transdifferentiation, and reprogramming efficiency depend on many unknown factors, such as cell identity, including cell cycle status, circadian, and epigenetics, as well as the heterogeneity of MEFs. By identifying the mechanisms involved in the process with small molecules, we will increase the induction efficiency. Therefore, we anticipate that modified methods for inducing human peripheral neurons and other neural crest derivatives using small molecules only will be developed in the near future to improve therapeutic approaches.

## Conclusion

Here, we performed a step‐by‐step treatment of small‐molecule cocktails to induce neuronal cells from somatic cells. We observed the generation of MAP2‐ and NeuN‐positive neurons from MEFs. Gene expression profiling revealed that the small‐molecule treatment induced neural crest‐like precursors; thus, the induced neurons were neural crest‐derived PNS neurons. Small molecules have significant advantages in terms of cell permeability, nonimmunogenicity, and ease of handling using standardized protocols. Thus, our induction method in mouse cells is a safe, promising approach that may contribute to the development of therapeutic applications in human cells. On the other hand, our step‐by‐step induction method includes the use of various epigenetic and signaling modulators, and its complex interactions. Thus, in the present, we cannot rule out the potent risks of various cell damages, senescence, or cancerization. Ensuring the safety and viability of induced cells with the use of other simplified combinations of small molecules will be required for further therapeutic applications.

## Author contributions

YT and YSK conceived the study. YT, TW, Y Saito, and YSK wrote the manuscript. YT and TW performed the experiments. HK and Y Shibuya prepared MEFs. HK performed the microarray experiments. YT, Y Saito, and YSK analyzed the microarray data. SS, WA, and HO contributed reagents and discussions.

## Supporting information


**Fig. S1**. Raw data for qPCR analysis of target genes in control and chemical‐treated MEFs. The expression value of each target gene was normalized to that of *U36b4*. *P < 0.05.

**Fig. S2**. Time series changes in the expression levels of the neural stem cell marker genes *Pax6* and *Sox2* in chemical‐treated MEFs. The expression level of each target gene was normalized to that of the control sample. For comparison, the mRNA expression levels in the adult mouse brain are also shown. n.d., not detected.

**Fig. S3**. Fluorescent images of GFP fluorescence (green), SOX10 (red), and cell nuclei (blue) in the chemically treated Wnt1‐Cre/EGFP MEFs at day 10. Scale bar: 100 μm.

**Table S1**. qPCR primer sequences.

**Movie S1**. Imaging of the calcium indicator fluo‐4 in the control sample for 100 s on day 21. Images were captured at a rate of 2 frames·s^−1^.

**Movie S2**. Imaging of the calcium indicator fluo‐4 in the chemical‐treated sample for 100 s on day 21. Images were captured at a rate of 2 frames·s^−1^.

**Movie S3**. Time‐lapse fluorescent imaging of control Nestin‐EGFP MEFs during the 70‐h period. Images were captured every 30 min.

**Movie S4**. Time‐lapse fluorescent imaging of chemical‐treated Nestin‐EGFP MEFs during the 70‐h period. Images were captured every 30 min.
