# Supplementary figures and images for: Brief exposure to small molecules allows induction of mouse embryonic fibroblasts into neural crest‐like precursors
Source: FEBS Lett. 2017 Feb 9;591(4):590–602. doi: 10.1002/1873-3468.12572 (PMC5347899; doi:10.1002/1873-3468.12572)

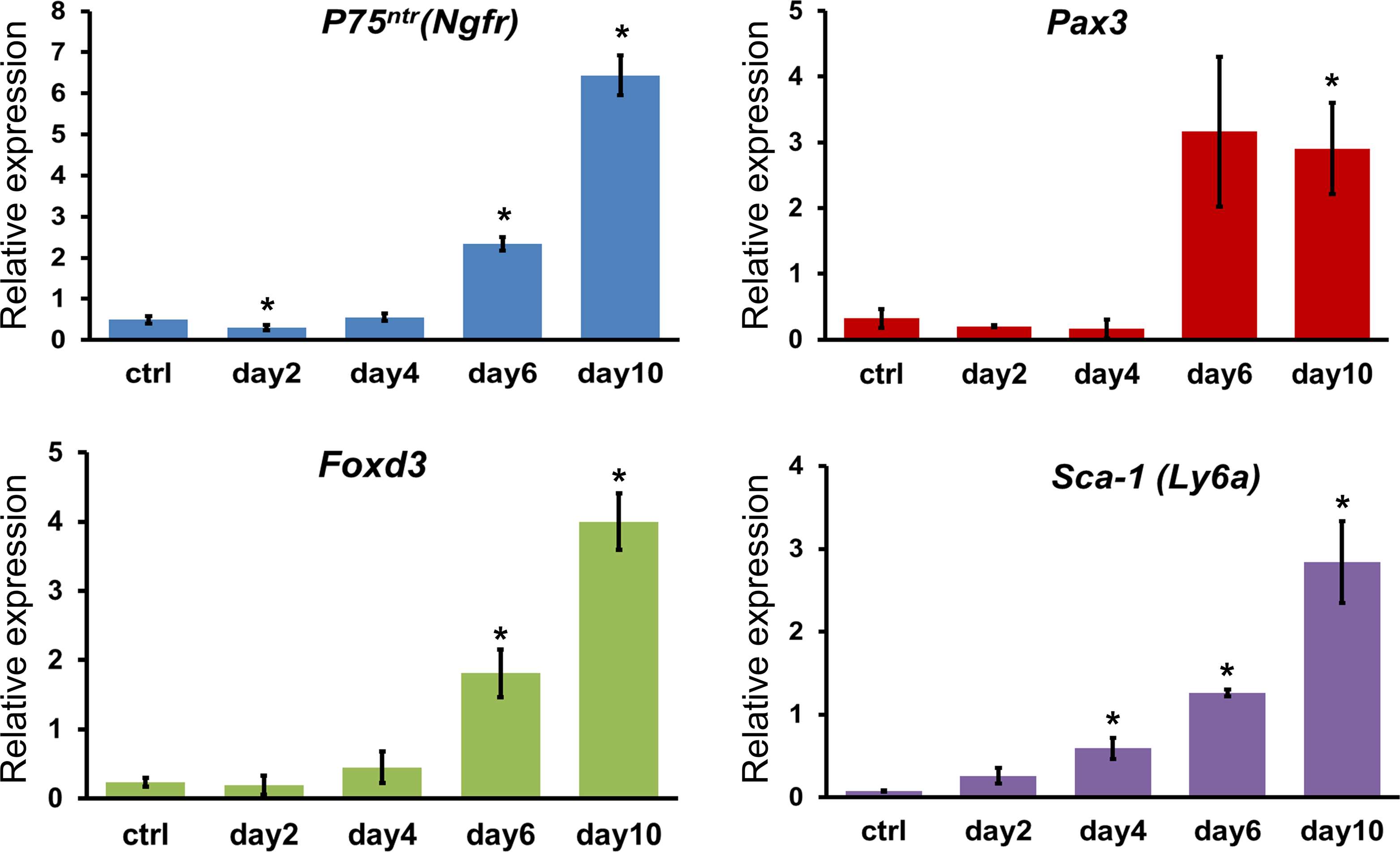

Supplement: Supplementary file 1 — Fig. S1. Raw data for qPCR analysis of target genes in control and chemical‐treated MEFs. The expression value of each target gene was normalized to that of U36b4. *P < 0.05. [file FEB2-591-590-s001.tif]

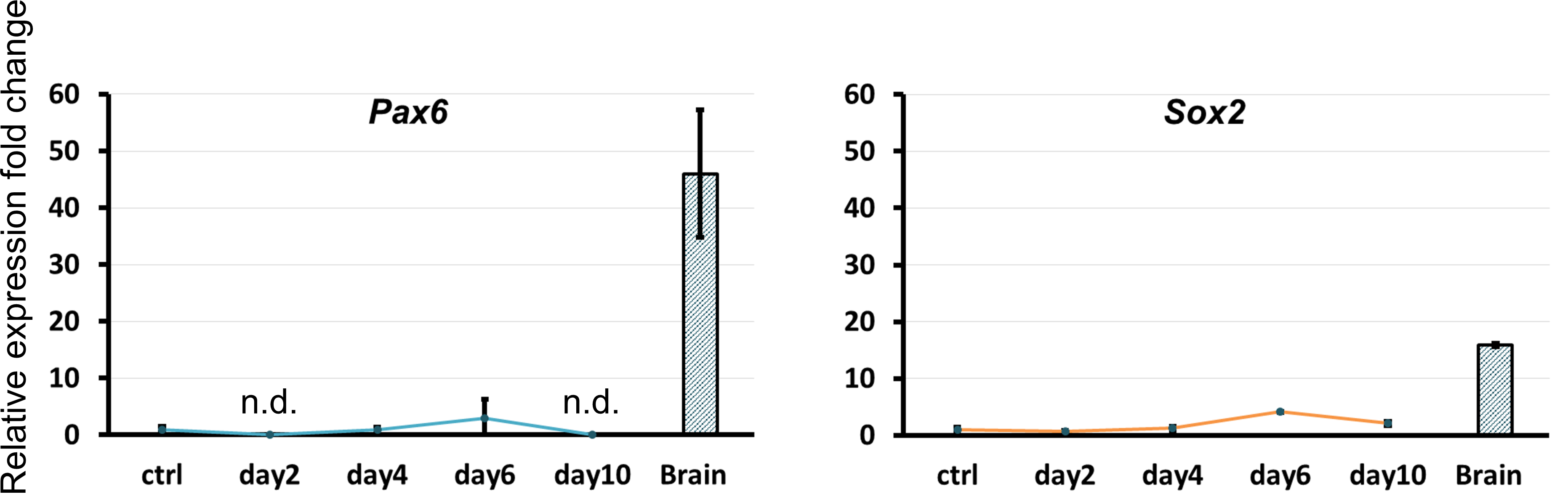

Supplement: Supplementary file 2 — Fig. S2. Time series changes in the expression levels of the neural stem cell marker genes Pax6 and Sox2 in chemical‐treated MEFs. The expression level of each target gene was normalized to that of the control sample. For comparison, the mRNA expression levels in the adult mouse brain are also shown. n.d., not detected. [file FEB2-591-590-s002.tif]

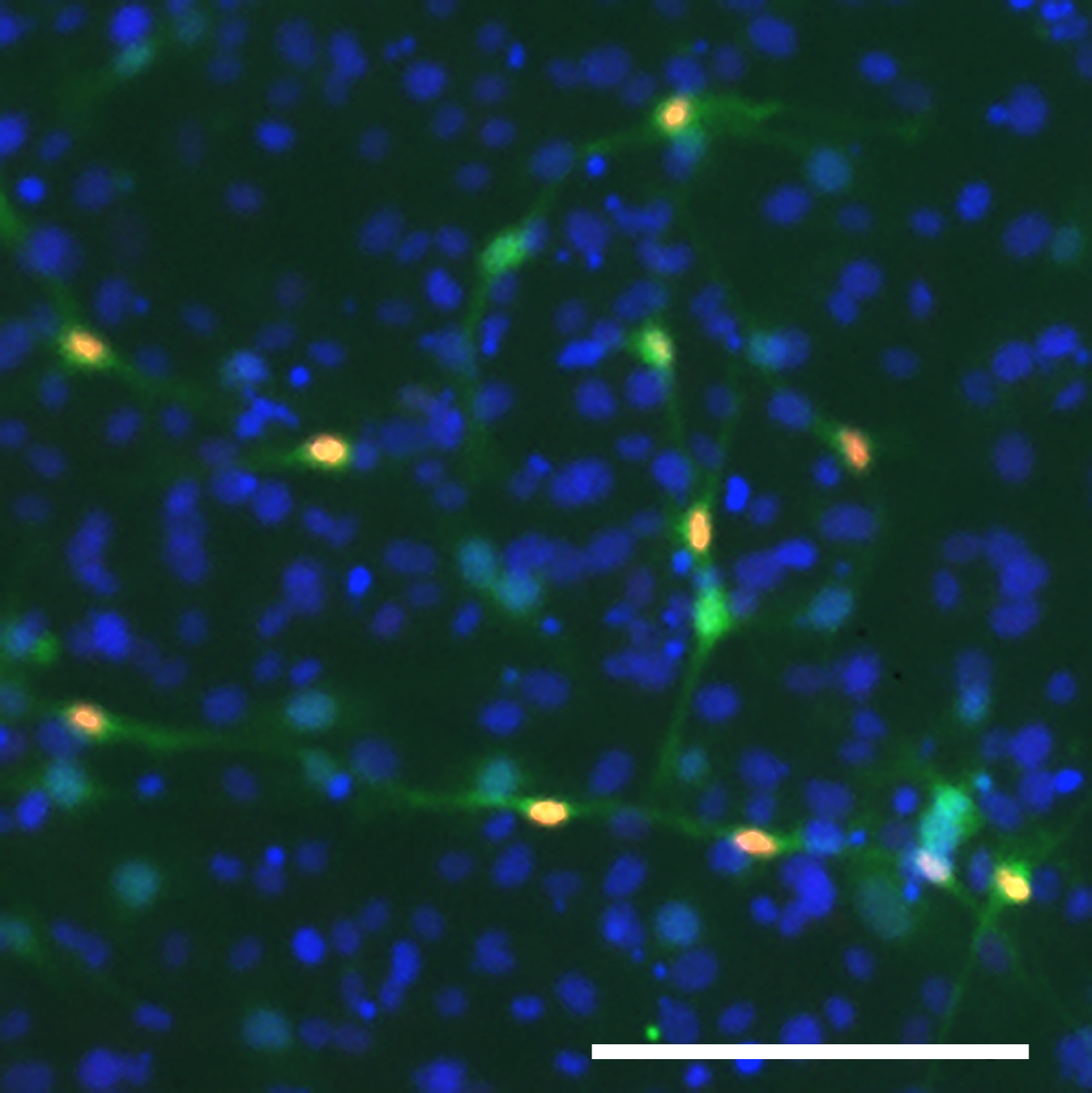

Supplement: Supplementary file 3 — Fig. S3. Fluorescent images of GFP fluorescence (green), SOX10 (red), and cell nuclei (blue) in the chemically treated Wnt1‐Cre/EGFP MEFs at day 10. Scale bar: 100 μm. [file FEB2-591-590-s003.tif]

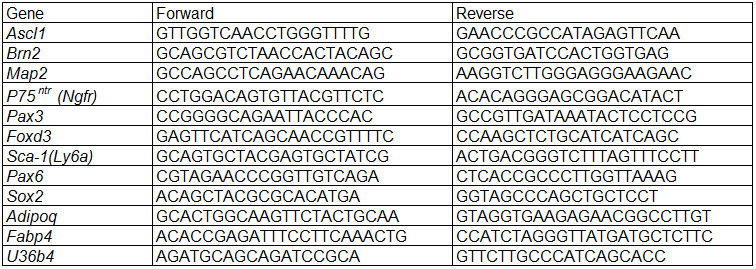

Supplement: Supplementary file 4 — Table S1. qPCR primer sequences. [file FEB2-591-590-s004.tif]
